# Congenital Occlusion of the Vagina

**Published:** 1853-04

**Authors:** 


*Congenital Occlusion of the Vagina.*—Throughout the medical journals are scattered notices of congenital occlusion of the vagina, sometimes combined with obvious arrest of development of the uterus,—sometimes with simply an imperfect condition of the vaginal orifice. We have upon former occasions mentioned the particulars of cases of extroversio vesicæ in the male, where the generative organs were incompletely formed: and we recall an observation made by Mr. Coote, that the arrest of development was not confined to the external parts, but that it extended to the whole segment of the body and the system of organs in wich these imperfect structures were situated. The same remark is applicable to congenital occlusion of the vagina. In some instances the nymphæ alone are adherent; in others, one, two, or more inches of the anterior part of the vagina are obliterated; the uterus may be ill formed, and the ovaries in no condition to mature ova, but in all cases the arrest of development of the internal parts is in relation with the amount of external deformity. The practical points connected with this law are equally applicable to the two sexes. In the male, suffering from extroversio vesicæ and fissured penis, the bones of the pelvis are usually small, and the pelvic cavity is shallow; the bladder, almost an abdominal viscus, retains its fœtal connexions to the peritoneum; the prostate gland is small and rudimentary; there is no trigon vesicæ uncovered by peritoneum; consequently we read without surprise of surgeons wounding the serous membrane with the trochar in their attempts to establish a rectovesical fistula as a preliminary step in the cure of this malformation. As regards the female, we may infer, that if with congenital occlusion of the vagina there be, at the time of puberty, no indications of the menstrual secretion, both external and internal organs are in a condition which cannot be relieved by surgery; but if the uterus, to all appearance, be healthy; if it become in course of time distended with menstrual secretion, and the patient suffer the usual pains and inconveniences, we may conclude that there is a vagina, an os tincæ, uterus, and ovaries, but that from some cause the external orifice, and an inch or more perhaps of the external meatus, are obliterated and adherent. It follows, then, that an operation, carefully performed, may relieve the patient of this distressing affliction; the vagina may be opened beyond the occlusion, and a canal may subsequently be established by the use of pessaries.

In the *New York Journal*, 1845, there is an account of a young German woman suffering from occlusion of the vagina. She had the sexual passion, but had never menstruated. Her general health was good. On inspection, it was found that she had no vagina. there was no abdominal swelling. Dr. Watson introduced into the urethra a silver catheter, which he committed to the charge of an assistant. Then passing the fore finger into the rectum, he divided the parts at the natural situation of the vagina, between the catheter and the finger. After dividing an inch and a half of tissues, the parts yielded to pressure, and the passage was restored to the os tincæ; it was, however, small, and the uterus was atrophied. The passage was kept open by the pessary, and ultimately rendered fit for all its functions by continued distension.

The *Medical Times*, 1845, contains some remarks upon this affection by Dr. Vaudroy; and Maissonneuve has performed an operation similar to that of Dr. Watson and of Mr. Wormald, who has lately successfully treated a case in most points similar to that which we have noticed above from the *New York Journal*.

Emma W., aged 19, a well formed and not bad looking girl, with an unmeaning and vacant expression of countenance, was admitted into St. Bartholomew's Hospital, Nov., 1852, under Mr. Wormald, with complete occlusion of the vagina. The labia, when open, seemed to bound a wall of mucous membrane, in which were seen both clitoris and urethral orifice, but there was no passage towards the uterus. The finger introduced into the rectum came in contact with a solid, elastic, bulging tumor, evidently the uterus distended by menstrual secretion, and pressing upon the anterior wall of the rectum. There was no apparent indication of a vagina, but the uterus bulged downwards to within about two inches of the surface of the perineum. The patient suffered considerable inconvenience from the pain in the back and loins at the menstrual periods; the bowels had become habitually costive. The rectum having been emptied by proper remedies, and the viscera being in a healthy state, Mr. Wormald performed the following operation, December 3: Chloroform having been administered, and the bladder and rectum previously emptied, the patient was tied, as in the operation of lithotomy. Mr. Wormald made an incision in the perineum, extending from the left labium obliquely downwards and outwards to the ramus of the ischium in the direction of the os tincæ, and in the interval between the urethra and the rectum, the coats of tl e latter viscus being indicated by the presence of the forefinger of the left hand introduced per anum. After carefully cutting in this narrow interval for about an inch and a half to two inches, Mr. Wormald came upon some yielding tissues, and then to the uterus. A trocar passed readily (and it was suspected through the os tincæ) into the cavity of the organ, and there was discharged fourteen ounces of thick, grumous, bloody fluid. A gum elastic catheter was introduced, and the patient was then removed to bed. There was an escape of bloody fluid during the next thirty-six hours, but this has slowly subsided. The patient has suffered occasionally from retention of urine, but there have been no unfavorable symptoms, and there is every prospect of a successful result.

Mr. Callender, the house surgeon, examined the fluid microscopically, and found that it consisted of epithelial scales, and altered blood discs.

There is reason to believe that, in the present instance, the vagina, which was obliterated to an extent of two inches from its orifice, yet existed above that spot, but was occupied by the distended uterus, which had sunk much nearer the perineum than natural, owing to its great enlargement.—*London Medical Times and Gazette.*

